# Older persons’ experiences regarding evaluation of their medication treatment—An interview study in Sweden

**DOI:** 10.1111/hex.12967

**Published:** 2019-09-26

**Authors:** Malin Holmqvist, Johan Thor, Axel Ros, Linda Johansson

**Affiliations:** ^1^ The School of Health and Welfare Jönköping University and Region Jönköping County Jönköping Sweden; ^2^ Jönköping Academy for Improvement of Health and Welfare the School of Health and Welfare Jönköping University Jönköping Sweden; ^3^ Institute of Gerontology. Aging Research Network‐Jönköping the School of Health and Welfare Jönköping University Jönköping Sweden

**Keywords:** attitudes, health knowledge, medication therapy management, older adult, patient participation, patient safety, practice, qualitative research, quality improvement

## Abstract

**Background:**

Older persons with polypharmacy are at increased risk of harm from medications, and this issue is a global patient safety challenge. Harm may arise at all stages of medication use and may cause hospital admission, additional resource utilization and lower patient satisfaction. Older persons’ participation in their own care may increase patient safety. Their views on the evaluation of their medication treatment, and their own involvement in it, are crucial yet poorly understood.

**Objectives:**

To identify opportunities to make the medication use process safer, we explored and described older persons’ experiences of evaluation of their medication treatment.

**Design:**

Semi‐structured interviews were performed with 20 community‐dwelling older persons (age 75‐91 years) in Sweden. Data were analysed using inductive qualitative content analysis.

**Results:**

These older persons reported having a responsibility to engage in their medication evaluations, although some felt unable to do so or considered themselves unconcerned. Evaluation, in their experience, was facilitated by continuity of care and an invitation to participate. However, some older persons experienced not receiving a comprehensive medication evaluation.

**Discussion and conclusion:**

Older persons want to be actively involved in their medication evaluations, and this may represent an underutilized resource in the pursuit of patient safety. Their trust in physicians to undertake evaluations on a regular basis, although that does not necessarily occur, may cause harm. Patient safety could benefit from a co‐production approach to medication evaluations, with health‐care professionals explicitly sharing information with older persons and agreeing on responsibilities related to on‐going medication treatment.

## 1 BACKGROUND

The World Health Organization (WHO) identifies ‘Medication without harm’ as the third global patient safety challenge.^1^ Harm from medications, often called adverse drug events (ADEs), occurs at all stages in the medication use process and cause hospital admission, additional resource utilization and lower patient satisfaction.^2^ In a review of studies concerning preventable ADEs in ambulatory care among community‐dwelling persons, the median prevalence rate was 16.5%, with a higher rate among older persons.^3^ This finding highlights the need for efforts to reduce ADEs, especially in older persons. Improving the medication use process, including prescribing, preparing, dispensing, administering, monitoring and evaluating, may reduce or prevent ADEs 4 For older persons, inappropriate polypharmacy is a leading cause of ADEs.^5^ Studies with different interventions to reduce inappropriate polypharmacy, such as medication review or assessment of medications, have had difficulties in demonstrating clinically significant improvements.^6^, ^7^ ADEs are common but often preventable in older persons in ambulatory settings. These events often originate in the prescribing or monitoring stage.^8^ Consequently, interventions aimed at these stages seem most beneficial in the prevention of ADEs. Traditionally, monitoring involves performing a physical examination and reviewing the patient's history and laboratory tests. An evaluation based on the results of this monitoring informs decisions about future treatment.^4^ Here we define ‘evaluation’ as an assessment of performance against an established set of goals or objectives at a point in time.^4^, ^9^, ^10^ Evaluation is continuously relevant, not only at fixed intervals, because medical conditions, especially for older persons, can change rapidly. Regulations by The National Board of Health and Welfare in Sweden direct prescribers to document plans for when and how a treatment will be evaluated.^10^

Older persons themselves, their relatives and health‐care professionals play significant roles in the medication use process.^11^ Persons participating in their own care may help reduce the risk of errors and increase patient safety, likely because they are involved, and vigilant persons can observe and communicate problems they experience before these problems result in adverse events.^12^, ^13^ While many older persons want to participate in their own care,^14^ not all have the desire or ability to do so. Older persons’ views on the evaluation of their medication treatment, and their own involvement in it, are crucial yet poorly understood. To generate opportunities for improvements to make the medication use process safer, it is important to know how these individuals currently experience the evaluation. Therefore, the present study explored and described older persons’ experiences of evaluation of their medication treatment.

## 2 METHODS

### 2.1 Recruitment and data collection

For this interview study, we recruited older community‐dwelling persons, with at least one chronic disease treated with medication(s) on a regular basis. We used the ‘older age’ definition, age 75 years or older, from the national regulation on medication use.^10^ To access a variety of experiences, a purposeful sampling was used and included persons with different numbers of diagnoses and medications, sex and age. Persons who managed their medications by themselves and those with support from a close relative were included. Persons who were not able to speak or understand Swedish and those diagnosed with dementia were excluded. Health‐care staff at five primary care centres in southern Sweden recruited the participants. Centres were selected to include large and small, publicly and privately run, centres. Staff verbally informed potential interviewees about the study and asked about participation. If the person wanted to participate, one author (MH) was notified and contacted the person within one week to give further information about the study. If the person agreed to participate, an interview was scheduled. All invited persons consented to participate.

One author (MH) performed all the semi‐structured individual interviews, guided by an interview guide that focused on the older person's experiences of evaluations of their medication treatment. The interview guide was developed based on knowledge on patient safety among the authors and guided by the medication use model 4 to cover different aspects of evaluation. It was pilot tested with three participants which yielded minor clarifying adjustments. The content of the pilot interviews did not differ from subsequent interviews, so these pilot interviews were included in the analysis. Each interview started with the invitation ‘Describe your latest appointment with a physician, in which you talked about your medication treatment’. The participants were then asked to share their experiences of how their medication treatment was evaluated, defined as ‘determining whether it is still adequate for you’, followed by questions addressing responsibility, feeling safe and the participant's own involvement in medication evaluation. Follow‐up questions to obtain a deeper insight into the participants’ experiences included ‘Can you please tell me more about this?’ or ‘Can you please explain this for me?’ At the end of the interviews, the persons were asked if they wanted to add something of relevance about evaluations of their medications that they thought was not addressed during the interview. Each participant was interviewed once between August 2017 and February 2018. To make the participants feel comfortable, they were asked to choose the time and place for the interview.^15^, ^16^ Most participants (n = 16) chose to be interviewed at home, and the others (n = 4) chose their primary care centre. The interviews were audio‐recorded and lasted between 28 and 65 (mean 47) minutes. After completing 20 interviews, no new information emerged, suggesting that sufficient data were collected to describe the phenomena. Therefore, no further participants were included.

Demographic characteristics were collected from the participants and from their medical record, with their consent (Table 1).

**Table 1:** Demographic and clinical characteristics of the participants

Gender (number)	
Female	9
Male	11
Age (y)	
Mean (range)	81 (75‐91)
Former profession (number)	
Administrator	2
Industrial worker	2
Craftsman	2
Farmer	3
Health‐care worker	5
Teacher	1
Technician	5
Medication management (number)	
Manage medication treatment alone	17
Manage medications with support from a relative or friend	3
Diagnosis at last yearly visit (number)	
Mean (range)	7.3 (3‐16)
Medication treatment, including ‘as needed’ (number)	
Mean (range)	12.7 (6‐26)

### 2.2 Data analysis

Two of the authors (MH and LJ) performed qualitative content analysis according to Elo and Kyngäs.^17^ As there is little previous knowledge in this area, the analysis followed an inductive approach.^17^, ^18^ First, the interviews were transcribed verbatim and read several times to become familiar with the data. Open coding involved writing headings in the margins and putting all headings together in a coding sheet. Data headings with similar content were compared and grouped together to generate subcategories. By abstraction, similar subcategories were formed into generic categories that were divided into main categories and reflected the content of the interviews. Examples of the analysis process are presented in Tables 2 and 3. Data were analysed and discussed among all authors until a consensus was reached. After data were analysed, the interviews were read through one more time to validate the categories that emerged. The preliminary result was then discussed with the other authors.

**Table 2:** Example of the analytical process—One generic category in the main category ‘Own role in the evaluation’

Meaning unit	Headings	Subcategories	Generic category
Yes, it must be the discharging doctor. ‐‐‐ Who else it is, I cannot understand. ‐‐‐ It can't be anyone else	Physicians are responsible for evaluation	Trust physicians to evaluate the medication treatment	Being unconcerned
I know nothing about this. I mean, you have to bow to the knowledge that the doctor has. You got to believe that, in some way, it helps	Cannot by myself so I trust my physician		
No, but I have a little in me that they know what to do. I trust them	I trust that physicians take the responsibility for continued needs		
But he didn't say anything last Friday. So it is ‘happy and pleased’. It must be	I assume everything is right if I hear nothing	Presume no information is good information	
It seems natural that they have some efficacy in the whole because they have prescribed it	Renewed prescription means continued treatment		
*And you should continue to eat them?* Yes, it seems so. He hasn't said anything else	I have no information of treatment length		
And then I got that medication. But the prostate is not enlarged, so I do not know why I should have it	I continue without knowing the desired effect		

**Table 3:** Example of the analytical process—One generic category in the main category ‘Views of evaluation received’

Meaning units	Headings	Subcategories	Generic category
Well, they have said… that it… it is good if you can stay under ten [blood sugar] ‐‐‐ One day we had nineteen and… I understand that it is not good	I receive information about target value	Receive information about planned evaluation	Being invited to participate
Yes it [the antihypertensive medication] is just for the high blood pressure. To keep it under control	I receive information about efficacy/ adverse reactions of treatment		
But they will keep track on me for five years now, with two blood samples every year. To see how it is then	I have knowledge about when and how treatment is monitored		
So he always asks what [blood‐pressure] I have at home. ‐‐‐Yes, and then I say as it is, that it is between 150 and 160	Physician shows interest in my own monitoring	Feel involved in the evaluation	
Yes, how I feel and ‐‐‐ and how I perceive it myself	Physician shows interest in my experience of the treatment		
The doctor, he said, if you have to get up more than twice a night, then we must try to do something about it	Physician asks me to contact healthcare in case of trouble		

## 3 RESULTS

The analysis of older persons’ experiences formed two main categories that described their *own role in the evaluation* and their *views of evaluation received*. Each main category included three generic categories.

**Figure 1:**
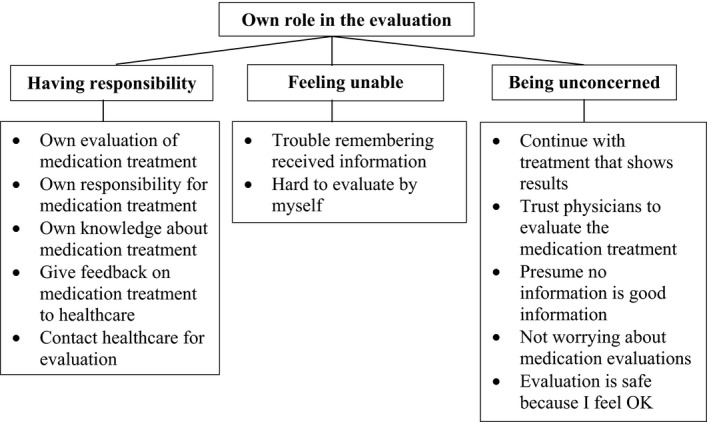
Main category and generic categories of ‘Own role in the evaluation’

The first main category, participants’ *own role in the evaluation* (Figure 1), included experiences of *Having responsibility* for the evaluation of their medications—seeking information and making appointments for regular visits with their physician; *Feeling unable*—finding it difficult to participate in the evaluation due to insufficient knowledge or lacking memory; and *Being unconcerned*—not worrying about on‐going treatment and therefore not about evaluations.

**Figure 2:**
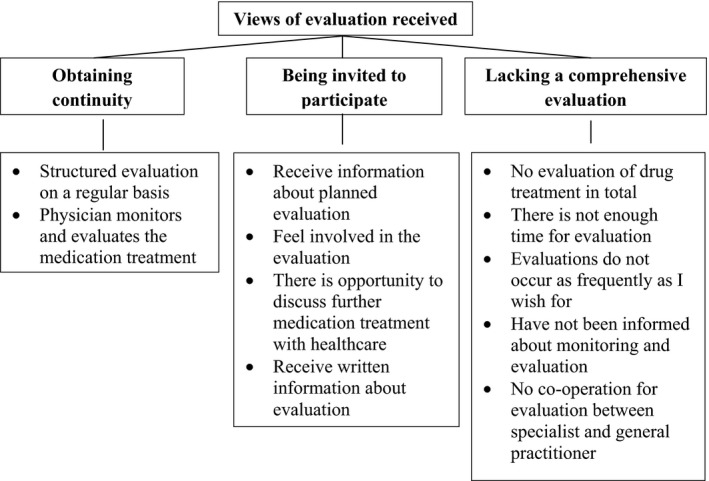
Main category and generic categories of ‘Views of evaluation received’

The second main category, participants’ *views of evaluation received* (Figure 2), included mixed experiences of *Obtaining continuity* in the medication treatment evaluation—for instance through regular visits to their physician; *Being invited to participate* in evaluations—when physicians talked about their medication treatment and asked about their experiences with it; and *Lacking a comprehensive evaluation* of their entire medication treatment—when no one seemed to take the overarching responsibility for the medication evaluation.

To help make sense of these findings, we elaborate on our analysis of the main and generic categories and present illustrative interview quotes.

### 3.1 Own role in the evaluation

#### 3.1.1 Having responsibility

‘Having responsibility’ reflected the older persons’ statements on having responsibility themselves, at least partially, for the evaluation of their treatment. Adhering to physicians’ instructions, primarily taking the medications as prescribed, to facilitate the physicians’ decisions on further treatment, emerged as the most important task. They also described how their responsibility for the treatment included making sure things worked out according to their understanding of the plan and, if not, giving feedback to health‐care professionals about side‐effects or other problems with the medications.

Yes, as I was about to say… The responsibility must be mine almost. That I alert them if it would fail. However, otherwise, the responsibility is there [with the physician] to do it. Because they have not told me anything else. (P11)

When prescriptions, often issued to last for a year (the legal limit in Sweden), expired, participants contacted their physician for prescription renewal. If an anticipated invitation for an appointment did not appear as expected, some persons reported contacting the physician's office to ask why and to schedule an appointment.

Participants referred to the physician as an expert, who gave them advice worth following, at their own discretion. If health‐care professionals had given no, or inadequate, information about the medication treatment, or if questions arose during medication treatment, the older persons searched for information on their own. For example, if suspecting a side‐effect, a friend or family member was asked for advice or information. Some older persons also referred to finding answers in the (mandatory) patient information leaflet included in the medication packaging. Having access to read their medical record online, which is widely available in Sweden, was described in a positive way, although the record was sometimes seen as difficult to understand.

Yes, sure, it's…I think it is great. ‐‐‐ I mean, when you visit the doctor again, you can look up ‘What did we come up with the last time?’ so to speak. ‐‐‐ Refresh the memory. ‐‐‐ Even if I keep my own record ‐‐‐ that is the official one [the on‐line medical record] ‐‐‐ Therefore, I think that is good. ‐‐‐ That is positive! Even if you cannot understand all the medical jargon that doctors use, that… we have to accept. We're not supposed to be able to know that ‐‐‐ or should need to know. (P11)

Taking actions and observing symptoms or adverse reactions provided the older persons a sense of control and made the medication treatment more interesting. They sometimes documented measurements, observations or planned visits in their own records. When monitoring medication effectiveness themselves, for example measuring their blood pressure, blood glucose level or merely observing symptoms, they considered it their duty to report findings to their health‐care professional. The older persons sometimes questioned the way evaluations were performed, if they did not feel confident with it.

#### 3.1.2 Feeling unable

The older persons described feeling unable to participate in the evaluation of their own medication treatment in several ways, including having trouble remembering verbal information about treatment goals, test results or how the treatment was supposed to be evaluated, especially if they received too much verbal information at one consultation. These memory challenges made them uncertain of whether they had received that information and what actions they were supposed to take.

Yes, some things one remembers ‐‐‐ but it can be like stuffing too much information in, so to say. When you sit and go through a list like this, you know, and you concentrate ‐‐‐there may be something that gets lost, you know. (P2)

The older persons sometimes found it difficult to interpret the medication effectiveness and potential side‐effects by themselves. Knowing whether the current treatment was the right treatment for them was difficult because they were unsure of how they would feel without the medications.

Previous experiences with adverse reactions led to insecurity about subsequent medication treatment. Similarly, not noticing any difference when medications were changed or the dose decreased created uncertainty of the treatment value. Sometimes, symptoms remained but the treatment was continued anyway. These experiences caused anxiety about medication treatment in ways that they sometimes found hard to address.

That is when, if you get side effects. Or you cannot notice it ‐‐‐ That one has received … too much? ‐‐‐ And one doesn’t know … Side effects, it may be so different with that. Because you may feel slightly strange. It may be for other reasons. ‐‐‐ So, it's not given that it's the drugs either ‐‐‐ That bothers me so. (P18)

#### 3.1.3 Being unconcerned

Being unconcerned about the evaluation reflected older persons’ experiences that taking medications was not stressful or worrying, but essential. Another reason for the lack of concern was related to age. Some persons accepted adverse reactions because of their age or because they thought that medications at their age were intended more to relieve symptoms than to cure the underlying condition.

I don´t experience it as stressful in any way. ‐‐‐ Or that I wonder why I have that [medication] or not. ‐‐‐ Yes, it's as I said, it's a fuel. (P5)

Additionally, being unconcerned about the evaluation was related to the older person's trust in the physician to have the knowledge needed to perform the task properly. As long as they were feeling fine and did not experience side‐effects or worsening of symptoms, they did not question the way the evaluation was performed and saw no need for monitoring visits. The older persons expressed that ‘no news is good news’ and presumed everything was fine if they did not hear anything. They expected that treatment was under control if they did not receive any information of concern about test results or directions to change the treatment.

And then I go and take blood tests. And I have never heard anything so it must be fine then. *[Interviewer] Well, but then… do they contact you and tell you that things are fine then?* No, no, they never do. ‐‐‐ However, I have worked it out… I understand. It’s only when, if there is anything that is wrong. Then, they will contact you. ‐‐‐ At once. Therefore, I trust that. (P3)

Feeling better after a medication had been initiated resulted in older persons not asking about the expected duration of treatment or the possibility of treatment cessation because they feared that changes in medication treatment could make them feel worse. Minor adverse reactions could be tolerated if the medication seemed to work well in other aspects. Although the intended duration of treatment in many instances was not known, it was not described as a cause for concern. A medication was sometimes continued without knowing why it was prescribed and without questioning its use. They interpreted a renewed prescription as a signal that the medication was evaluated and considered appropriate for another period of time, even if it had initially been intended for a finite period.

### 3.2 Views of evaluation received

#### 3.2.1 Obtaining continuity

Obtaining continuity of care with a structured evaluation of their condition and treatment on a regular basis, for example via a yearly visit to their general practitioner (GP), was appreciated and facilitated the medication evaluation. Typically, older persons received written invitations to these visits, and the invitations were expected. They emphasized that seeing the same physician, who knew them and their medical history, resulted in a sense of continuity and safety.

Yes, but it's as different as night and day [to see the same physician instead of different ones] ‐‐‐ because then you can just pick up where you left off ‐‐‐ instead of having to go over everything from the beginning, everything that has happened and so on. (P14)

The older persons reported several actions taken by physicians to evaluate their medications, including reconciliation of their medication list (ie creating an accurate list of medications that the patient is taking), performing a physical examination and ordering laboratory tests. Recurrent examinations and tests created a feeling of familiarity with the procedures. Another route to this familiarity was when nurses, for example at a diabetes clinic, were involved in regular evaluations because the nurse was seen as a positive bridge between the individual and the physician.

She [the diabetes nurse] and the doctor have very much in common, or talk, talk it through [the medication treatment]. ‐‐‐ So that she knows what the doctor thinks and so on… So that… I think that is great! (P4)

#### 3.2.2 Being invited to participate

When a physician or nurse invited them to discuss their medication treatment, the older persons felt safe and involved. Having a dedicated person to contact at a primary care or specialist care centre made it easier to ask questions or request an appointment for an evaluation.

Being involved in the evaluation of medications was experienced when the physician shared results from blood tests, blood pressure readings, diagnostic imaging or other tests and gave advice on continuing medications. Receiving information from health‐care professionals about the purpose, expected benefits and potential adverse reactions and plans for medication monitoring were similarly helpful and made them feel like a partner in their own treatment. Written information, for example a printed list with current medications, including their indication, made it easier to participate in further treatment. One person shared a positive experience of being involved in the plan for evaluation:

Now, now that I got this plan from the doctor’s office on how they intended to do it. ‐‐‐ It is the first time they have reported what they have been thinking ‐‐‐ And how they have planned to manage it. Otherwise, it is just that, you get a new appointment and you get better. Bye! Send them off to home! Then after a while, you are back. ‐‐‐ However, that … so now I think they are on the right track that they've decided what to do. *[Interviewer] And you have received a plan too so you know about it?* Exactly. Yes, but I know what is expected to happen. ‐‐‐ And I know that something is being done, and not only for getting through that weekend. (P20)

When physicians asked for the older persons’ views or experiences regarding their medications, for example their own blood pressure readings taken at home, they felt involved in the evaluation and experienced that the treatment worked well. Being encouraged to monitor and adjust doses at home between visits based on their own monitoring increased their involvement in the treatment and created a feeling that the physician trusted them. When experiencing problems with the treatment, the older persons felt welcome to contact their physician's office.

Because I know that when I got blood pressure medications then, ‐‐‐ then the doctor said ‘If there is anything that you feel then, that you have not felt before, because you are taking this medication, you will have to let us know’ ‐‐‐ But I have never felt that. (P9)

#### 3.2.3 Lacking a comprehensive evaluation

Older persons sometimes expressed concerns about the lack of a comprehensive evaluation of all their medications taken together. Those who received prescriptions from several physicians missed having someone explicitly evaluate whether the mix of all medications was appropriate, and whether the purposes of the prescribed medications were achieved. Sometimes, they felt that the effects of some medications opposed those of other medications.

Well, but it's not easy to know. They [the physicians] say, some say ‘Yes, I have to have a pill to calm me down’. Yes, yes, then they will get one. ‘I must have one that perks me up’ ‐‐‐ Yes, then they get one for that. And then, they don’t know which is which ‐‐‐ or whether they work or not…’ (P18)

Older persons who had contact with other physicians in addition their GP described a lack of cooperation between them. For example, medication initiated by a medical specialist was later evaluated by the GP on referral, but the GP was not familiar with the treatment, and it was unclear to the older person who was responsible for the evaluation. Knowing who to ask for further evaluation sometimes seemed difficult because physicians rotate when they are under training or medical locums.

The older persons questioned the practice when physicians renewed their prescriptions without an appointment, because the physician may lack up‐to‐date information about medication effectiveness, for example a recent blood pressure reading. Some participants stated that visits lacked sufficient time, and it was difficult to secure time to discuss the treatment with the physician, especially its purpose and potential side‐effects.

No, no, nothing, but she said ‘You should take this pill’ and I had no idea what it was. ‐‐‐ She could have sat down [bedside] and said that this one is for this and that and so on, but no… ‐‐‐ It’s not… they do not have time for that. (P14)

A lack of information about how, when and by whom treatment was supposed to be evaluated was expressed. Even if health‐care professionals sometimes invited the older persons to monitor their treatment themselves, the results were not always requested. When receiving results, for example from laboratory tests or diagnostic imaging, often no findings were specified; they were just told ‘everything is fine’, although the older persons wanted to receive more specific information.

Yes of course. If you go and take blood tests, then you ought, at least, to get some kind of result, one would think. ‐‐‐ Because the doctor, he gets the test results, but I do not. (P18)

The frequency of monitoring and evaluation varied over time and between different treatments. When medications were initiated or changed, frequent evaluation with visits or phone calls was received, but medications were not evaluated as frequently over time. For some older persons, years passed between assessments, which they questioned. They reasoned that this lack of evaluation was related to their age, that it may not be needed or that treatment monitoring was omitted for older persons simply because they were older.

I asked the doctor on my recent visit ‘Aren’t you going to check my bones’ I said. ‐‐‐ However ‘No,’ she said, ‘they had not said anything from there [the hospital]’. ‐‐‐ You know, when you get old, they withdraw all such assessments. (P14)

## 4 DISCUSSION

### 4.1 Reflections on the findings

The older persons experienced their medication treatment as safe, but they wished that a designated health‐care professional took the overarching responsibility for their medication evaluations. Their trust in the physicians and their desire to be involved in their medication treatment are two important findings that must be addressed. From a safety perspective, it is important to identify and strengthen actions that make patients feel safe but also ‘remain sensitive to possibilities of failure’, so failures are detected and addressed before they become ADEs.^19^

The older persons revealed a deep trust in physicians and assumed that these physicians took responsibility for the evaluation of their medications. This trust in physicians’ medical knowledge on whether to proceed or discontinue a medication mirrored findings about older persons’ attitudes towards de‐prescribing.^20^ If older persons do not question the evaluation of their medications because of a deep trust, health‐care professionals must be aware of the impact of this trust and remain alert to risks that may occur due to naiveté about medication safety. Even among older persons who experienced the treatment as safe, several desired a comprehensive review of all of their medications, since, in their view, no one had that overarching responsibility for their medications.

Lack of evaluation was sometimes considered as unimportant because of their age. The older persons felt that it was not worthwhile to evaluate their treatment simply because they were older. These views, for example to expect to have many medications or side‐effects simply because you are older, were noted previously by older persons themselves and health‐care professionals.^21^ To view medication treatment as part of life and not worry much about regular medication evaluations, as long as one feels all right, echoes findings from interviews with older persons in New Zealand on attitudes to medications.^22^ This circumstance may lead older persons to not question why evaluations are lacking and can be a safety concern. Older persons’ deep trust in the physicians, combined with a lack of concern about risks with their medication treatment, may make them less prone to notice and report potential safety hazards. To prevent inappropriate polypharmacy and ADEs in older persons, these findings highlight the importance to take a systematic approach to assess and improve medication treatment, which requires cooperation between health‐care professionals and patients.^23^

This study shows that there are older persons who want to be involved in the evaluation of their medications, just as in care in general.^14^ However, it also revealed several challenges to patient involvement, including insufficient time at appointments to discuss on‐going treatment, difficulties in understanding or remembering information, and lack of written information. This corresponds well with factors found to make older persons feel insecure about their medications.^24^ In Australian nursing homes, residents who lacked understanding of the purpose of their treatment, or risk of potential ADEs, tended to develop apathy towards the risks related to polypharmacy.^25^ In a recent survey, people in Sweden reported having less time for consultation with a physician and receiving less information about their treatment compared with people in other countries.^26^ Sufficient consultation time, written information and good access to health‐care professionals to ask questions are important considerations to ensure that older persons understand how their medication treatment is supposed to be evaluated, which is important for their ability to participate and enhance safety.

Persons who saw themselves as responsible for their medication evaluation monitored their treatment at home and proactively scheduled follow‐up appointments with their physician. To be involved and self‐reliant in the medication use process can, as shown in studies of self‐administration, make people feel safe.^27^, ^28^ Giving older persons support to be more active in their health care, for example during transitions such as discharge from hospital and discussing symptoms and adverse reactions to be alert to, may reduce the risk of undesired consequences such as readmission.^29^ An active partnership between physicians prescribing medications and the person taking them (or not) exemplifies the co‐production of health and health care.^30^, ^31^ However, there are differences in older persons’ perceptions, ability and motivation regarding their involvement in the medication evaluation, which may be challenging to health‐care professionals.^32^ Therefore, it is important that health‐care professionals regularly assess whether an older person, or any patient, remains capable of managing all parts of the medication use process or needs further support. Support should be tailored to each person's understanding of their health and medications, and their ability to manage them. Health‐care professionals can address this variation by adapting their support and regular follow‐up visits in a person‐centred manner.^33^, ^34^

From a patient safety perspective, knowledgeable and engaged older persons may help prevent ADEs, perhaps more often than is typical today.^35^ Health‐care professionals could tap into this resource for added safety by enabling older persons to take a more active role in the evaluation. For example, physicians can share the plan for medication treatment, including its intended effects, potential adverse reactions, timing of evaluations and aspects to observe. Now that patients in Sweden, as in many other countries, can access much of their medical record online, they have expanded opportunity to review information about their medication treatment at any time, not only during office hours. A coherent documented medication plan, co‐created and shared with the older person, might be a good way of guiding their further treatment and evaluation.

### 4.2 Methodological considerations

The major strength of this study is that it accessed the experience of medication treatment and evaluation in older persons, in depth, using semi‐structured individual interviews. To promote the trustworthiness of the study, we considered the credibility, dependability, confirmability and transferability of the data collection and analysis.^36^ One author (MH) performed all interviews, which promoted consistency across interviews. We used purposive sampling to access a range of relevant experience among the participants and achieve transferability. Primary care centres identified suitable participants on the authors’ behalf. We note that 25% (n = 5/20) of the participants had worked in health care, as nurse assistants, nurses or pharmacists. This experience may have provided these persons with a greater understanding of the medication use process than among older persons in general. This experience also likely provided these participants a good ability to discuss medication evaluation.

Selection of sampling size is important to ensure credibility.^37^ A preliminary analysis of 20 interviews grouped the data and created concepts, and therefore, we accepted the number of collected interviews. To achieve dependability in selecting relevant meaning units and form categories that covered the data, two authors (MH and LJ) analysed the material together. They then discussed preliminary results together with the other authors, combining perspectives from pharmacy, nursing, gerontology, medicine, patient safety, improvement research and qualitative methods to reach consensus. The inclusion and exclusion criteria and the purposive sampling method created dependability in the recruitment process.^37^ After the categories emerged, all interviews were read through one more time to ensure the confirmability of the participants’ experiences of evaluation.

## 5 CONCLUSION

Older persons’ experiences of the evaluation of their medications reveal several opportunities to improve medication treatment safety. Many older persons can and want to be actively involved in their medication evaluation, and this desire may represent an underutilized resource in the medication use process. However, their trust in physicians to undertake evaluations on a regular basis, although that does not necessarily occur, may cause harm. From a patient safety perspective, it appears that older persons will benefit from a co‐production approach to medication evaluations, with health‐care professionals explicitly sharing information with them and agreeing on responsibilities related to on‐going medication treatment.

## CONFLICT OF INTEREST

MH is employed in Region Jönköping County where the study was conducted and received grants from the Academy for Healthcare in Region Jönköping County for time to conduct the study. AR is employed in Region Jönköping County where the study was conducted. JT, AR and LJ have conducted this research as advisors to MH in her research training.

## ETHICAL APPROVAL

The Regional Ethical Review Authority in Linköping, Sweden, approved the study (#2017/292‐31), which adhered to the Declaration of Helsinki. The director of each primary care centre signed an approval for the primary care centres to participate in recruiting persons. A risk in enlisting health‐care staff to recruit participants may be that older persons feel obligated to participate. To diminish that risk, health‐care staff and the interviewer emphasized that participation was voluntary and that an older person's choice not to participate, or participants’ answers to interview questions, would not affect their usual treatment. Participants gave written consent before the interview started. All data were de‐identified to maintain confidentiality. Data are presented in a manner to avoid identification and protect the privacy and integrity of individuals.
